# Comparative Analysis of Adverse Event Profiles Among Seven Statins for Hypercholesterolemia Management Using the United States FDA Adverse Event Reporting System

**DOI:** 10.7759/cureus.81260

**Published:** 2025-03-26

**Authors:** Toru Ogura, Chihiro Shiraishi

**Affiliations:** 1 Clinical Research Support Center, Mie University Hospital, Tsu, JPN; 2 Pharmacy Department, Mie University Hospital, Tsu, JPN

**Keywords:** cardiovascular risk, database, drug safety, multiple comparisons, personalized medicine, pharmacoepidemiology

## Abstract

Background

Statins are fundamental in hypercholesterolemia management, with seven primary drugs available: atorvastatin, simvastatin, rosuvastatin, pravastatin, lovastatin, fluvastatin, and pitavastatin. While sharing a common mechanism of action, these statins exhibit variations in pharmacokinetic (what the body does to the drug) and pharmacodynamic (what the drug does to the body) properties (e.g., lipophilicity, cytochrome P450 metabolism), which may influence their safety profiles. Adverse events (AEs) such as myopathy and hepatotoxicity vary across agents, complicating clinical decision-making. The guidelines lack robust comparisons of all seven statins' AE profiles, presenting challenges for clinicians in balancing potency and tolerability.

Objectives

This study aimed to comprehensively compare the AE patterns and safety profiles of the seven statins in hypercholesterolemia management through a retrospective analysis of the United States Food and Drug Administration Adverse Event Reporting System (FAERS) database. By focusing solely on hypercholesterolemia, we aimed to control for potential confounding factors, providing a more focused comparison of statin safety profiles.

Methods

We conducted a retrospective analysis using data from the FAERS between 2004 and 2024. To control for confounding factors, this study included only patients prescribed statins with a clearly documented indication of hypercholesterolemia management. Patients prescribed statins for other indications or with missing data on the reason for prescription were excluded. Comparative evaluations employed the reporting odds ratio (ROR) and adjusted ROR (aROR), which were chosen for their efficiency in ease of interpretation, ability to adjust for confounding factors, and compatibility with statistical testing frameworks. This compatibility allowed for rigorous multiple comparison analysis, where each statin was sequentially set as a reference in 21 pairwise comparisons. To address the multiple comparisons problem, this study applied the Bonferroni correction, adjusting the significance level to 0.05 / 21 = 0.0024. Patient background variables were used as adjustment factors for the aROR. AEs were classified into 10 categories based on their characteristics.

Results

With atorvastatin as the reference, five statins (simvastatin, rosuvastatin, pravastatin, fluvastatin, and pitavastatin) demonstrated both significant ROR > 1 and aROR > 1 for gastrointestinal disorders. Conversely, five statins (simvastatin, rosuvastatin, pravastatin, lovastatin, and pitavastatin) demonstrated both significant ROR < 1 and aROR < 1 for metabolic disorders. When other statins were set as the reference, no consistent pattern of exclusively significant ROR > 1 and aROR > 1 or significant ROR < 1 and aROR < 1 was observed across all AE categories. Instead, a heterogeneous distribution of outcomes was evident. These results indicate that the patterns of AEs differ for each statin.

Conclusions

This study reveals distinct AE profiles among seven statins, providing critical insights to guide personalized treatment strategies. By aligning patient risk factors with specific statin AE profiles, clinicians can implement more targeted approaches to minimize AEs, potentially improving adherence and treatment efficacy. These findings directly inform clinical decision-making, enabling healthcare providers to optimize statin selection and management for individual patients.

## Introduction

The global prevalence of hypercholesterolemia has been steadily increasing, largely attributed to lifestyle changes accompanying worldwide economic development [1]. This trend poses significant public health implications, as elevated cholesterol levels are known to correlate with increased incidence of severe events, such as myocardial infarction, coronary artery disease, and mortality. In response to this growing health concern, statin therapy has emerged as a cornerstone in hypercholesterolemia management. Statins, a class of 3-hydroxy-3-methylglutaryl-coenzyme A (HMG-CoA) reductase inhibitors, have revolutionized treatment since the United States Food and Drug Administration (FDA) approval of lovastatin in 1987 [2]. These drugs function by competitively inhibiting HMG-CoA reductase, the rate-limiting enzyme in cholesterol biosynthesis, thereby reducing intracellular cholesterol levels and upregulating low-density lipoprotein (LDL) receptor expression [3]. This mechanism of action results in a significant reduction of serum LDL cholesterol (LDL-C) levels, which has been consistently associated with a decreased risk of cardiovascular events [4]. Beyond their lipid-lowering effects, statins have demonstrated pleiotropic benefits, including anti-inflammatory properties and improvement of endothelial function. These additional effects may contribute to their overall cardiovascular risk reduction. While the clinical relevance of these pleiotropic effects is still being investigated, some studies suggest they may lead to improved cardiovascular outcomes beyond LDL-C reduction alone. For instance, the anti-inflammatory effects of statins, as measured by reductions in C-reactive protein levels, have been associated with decreased cardiovascular event rates independent of LDL-C lowering. The statin family has expanded to include pravastatin, simvastatin, fluvastatin, atorvastatin, rosuvastatin, and pitavastatin, each with distinct pharmacokinetic and pharmacodynamic properties. These variations, particularly in lipophilicity and cytochrome P450 (CYP) metabolism, can influence their efficacy and safety profiles [5]. For example, lipophilic statins may have greater tissue penetration, potentially affecting both their efficacy in non-hepatic tissues and their side effect profile. Additionally, differences in CYP metabolism can impact drug-drug interactions, an important consideration in patients with multiple comorbidities. A comprehensive comparison of these characteristics is provided in Appendix A. While statins are generally well-tolerated, they have been associated with a range of adverse events (AEs) that can affect multiple organ systems [1]. Common AEs include muscle-related side effects such as myopathy, myalgia, myositis, and in rare cases, rhabdomyolysis [6]. Importantly, these muscle-related AEs have been observed to be dose-dependent, with higher statin doses generally associated with an increased risk. Furthermore, the incidence and severity of these AEs may vary among different statins, potentially due to differences in their pharmacokinetic properties. Other potential AEs encompass liver dysfunction and cognitive side effects. Nevertheless, various studies indicate that the incidence of serious AEs is relatively low, suggesting that for many patients, the cardiovascular benefits may outweigh the potential risks [7,8]. However, the balance of benefits and risks can vary depending on individual patient factors and should be carefully considered in each case.

Given the variations in efficacy, safety profiles, and potential pleiotropic effects among different statins, there is a critical need for comprehensive comparative studies to guide optimal statin selection in clinical practice. Previous studies of statin safety utilizing data from the FDA Adverse Event Reporting System (FAERS) [9] have often focused on specific AEs [10,11], potentially limiting their applicability to clinical decision-making. Clinicians typically consider a comprehensive profile of potential AEs when selecting a statin, rather than basing their decision on a single type of AE.

The current gaps in knowledge regarding the comparative safety profiles of different statins have led to challenges in clinical decision-making. For instance, the lack of head-to-head comparisons of all seven statins in terms of their comprehensive AE profiles has made it difficult for clinicians to personalize statin therapy based on individual patient risk factors. Current guidelines tend to discuss the efficacy and safety of statins as a class, without sufficiently comparing individual statin AE profiles [4]. This approach overlooks potential differences in AE patterns and frequencies that may arise from variations in pharmacokinetic properties among statins, such as lipophilicity and metabolic pathways. This gap has sometimes resulted in a "trial and error" approach to statin selection, potentially leading to unnecessary AEs or suboptimal treatment outcomes.

This study aimed to address the gap between clinical needs and previous studies by examining AEs associated with the seven statins in hypercholesterolemia management through a retrospective analysis of the FAERS database between 2004 and 2024. Employing multiple comparison analysis, a robust statistical method for pairwise comparisons, the study provided insights into AE patterns across the statin class, offering a nuanced perspective on their relative safety profiles. By focusing exclusively on statins for hypercholesterolemia, we controlled for potential confounding factors from diverse indications, although residual confounders, such as body mass index and comorbidities, inconsistently reported in FAERS, remain a limitation. Additionally, the lack of detailed clinical data, including lipid profiles and liver function tests, restricted a comprehensive assessment of disease severity and metabolic influences on AE occurrence. Recognizing these limitations, the study emphasized hypothesis generation over direct clinical application and highlighted that its findings should guide future research and pharmacovigilance while being contextualized within established clinical guidelines and patient-specific considerations.

## Materials and methods

Data source

This study utilized the FAERS database, a de-identified repository reported quarterly since 2004. The system transitioned from the AERS in the first quarter of 2004 (2004Q1) to the more comprehensive FAERS in 2012Q4. On January 31, 2025, we accessed the AERS and FAERS data files (designated as aers_ascii_yyyyQq.zip and faers_ascii_yyyyQq.zip, where yyyy indicates the year and q represents the quarter) from the official FAERS website. To ensure consistency, continuous variables were summarized as median with first and third quartiles. Categorical variables were summarized as frequency with reporting proportion (RP) [12], calculated as RP = (number of patients reported in the category of interest) / (total number of patients reported receiving a particular statin) × 100. Comparative evaluations were conducted using the reporting odds ratio (ROR) [13] and adjusted ROR (aROR), with each statin sequentially set as the reference in 21 pairwise comparisons. The significance level was adjusted using the Bonferroni method, resulting in a threshold of 0.05 / 21 = 0.0024. Therefore, a p < 0.0024 was considered statistically significant. To correspond with the adjusted significance level [14], an adjusted significance level of α = 0.0024. The corresponding confidence level is calculated as (1 - α), which equals 1 - 0.0024 = 0.9976, or 99.76%. This approach ensures consistency between the statistical hypothesis test and the confidence interval (CI) interpretation. Specifically, when a result is statistically significant, the 99.76% CI will not include 1. Three patient background variables of (sex), (age), and (reporter_country) were used as adjustment factors for the aROR. However, (wt) was not included as an adjustment factor due to a substantial amount of missing data. Patients with missing data for any of these three variables were excluded from the aROR calculation. While methods for imputing missing data exist, the FAERS database provides a limited number of patient background variables, making high-accuracy imputation challenging. Given that imputation with limited information could introduce bias, we opted for exclusion rather than imputation to maintain data integrity. The reference categories were set as female for (sex) and other countries for (reporter_country). Given that the United States contributed the highest number of reports, the variable (reporter_country) was treated as a binary variable (United States vs. other countries) in both univariate and multivariate analyses. It is important to note that the FAERS database only contains records of reported AEs and does not include data on instances where no AEs occurred. As a result, it was not possible to calculate the true incidence rates of each AE. This limitation may have a greater impact on ROR and aROR when comparing statins in which a larger proportion of patients did not experience AEs with statins in which a smaller proportion did not experience AEs. To distinguish our methods from conventional statistical techniques, we prefixed "reporting" to the names of our statistical analysis methods, consistent with established practices in prior studies. All statistical analyses were performed using software R version 4.4.1 (R Foundation for Statistical Computing, Vienna, Austria).

Patient background

**Figure 1:**
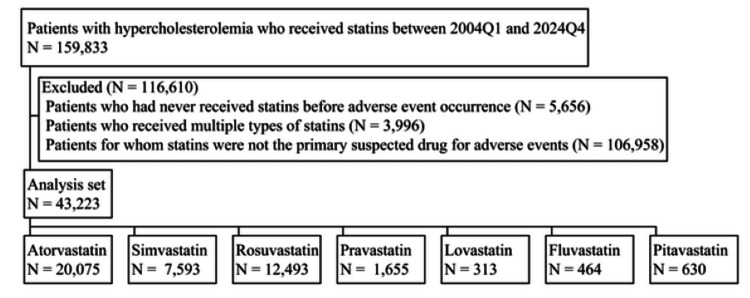
Flowchart of patients with hypercholesterolemia who received statins

We analyzed FAERS data between 2004Q1 and 2024Q4, identifying 159,833 patients with hypercholesterolemia management who received statins. After applying our exclusion criteria, 116,610 patients were removed from the initial cohort, resulting in a final analysis set of 43,223 patients. The distribution of patients across the seven statins in the analysis set is illustrated in Figure 1, with the following breakdown: atorvastatin (N = 20,075), simvastatin (N = 7,593), rosuvastatin (N = 12,493), pravastatin (N = 1,655), lovastatin (N = 313), fluvastatin (N = 464), and pitavastatin (N = 630). Table 1 provides a comprehensive summary of patient background variables for each statin. For all statins, except simvastatin, the number of female patients surpassed that of male patients with known sex. However, the high RP for unspecified sex data in pitavastatin cases suggests that, if these unknown cases were to be identified, there remains a potential for altering the relative magnitudes of RPs between males and females. Nevertheless, considering the known sex data for pitavastatin, where the RP for female patients was 37.5 compared to 23.2 for male patients, the likelihood of such a reversal appears minimal. The median age ranged from 64 to 68 years, with minimal differences observed among the seven statins. Similarly, when comparing the first quartile ages across the seven statins, the differences were negligible. The same pattern of minimal variation was observed for the third quartile ages among the seven statins. Regarding weight, the RP of unknown was relatively high for all seven statins. The median values for most statins ranged from 75 to 79 kg, with fluvastatin (68.0 kg) and pitavastatin (72.2 kg), showing lower median weights. With the exception of fluvastatin and simvastatin, the majority of AEs for all statins were reported from the United States.

**Table 1:** Summary of patient background Age and weight are summarized as median and first and third quartiles. Other data are summarized as frequency (RP). Q1: first quartile; Q3: third quartile; RP: reporting proportion

Items	Atorvastatin	Simvastatin	Rosuvastatin	Pravastatin	Lovastatin	Fluvastatin	Pitavastatin
	N = 20,075	N = 7,593	N = 12,493	N = 1,655	N = 313	N = 464	N = 630
Sex							
Female, n (RP)	11,205 (55.8)	3,433 (45.2)	7,253 (58.1)	902 (54.5)	179 (57.2)	277 (59.7)	236 (37.5)
Male, n (RP)	8,026 (40.0)	3,664 (48.3)	4,794 (38.4)	666 (40.2)	129 (41.2)	180 (38.8)	146 (23.2)
Unknown, n (RP)	844 (4.2)	496 (6.5)	446 (3.6)	87 (5.3)	5 (1.6)	7 (1.5)	248 (39.4)
Age, years							
Median	64.0	65.0	65.0	66.0	64.0	68.0	68.0
Q1-Q3	56.0-72.0	57.0-73.0	56.0-73.0	58.0-74.0	55.0-72.0	58.0-76.0	59.0-74.0
Unknown, n (RP)	4,378 (21.8)	1,395 (18.4)	2,080 (16.6)	336 (20.3)	65 (20.8)	83 (17.9)	295 (46.8)
Weight, kg							
Median	77.0	78.0	76.2	75.0	78.3	68.0	72.2
Q1-Q3	66.2-88.5	67.3-89.1	65.0-88.5	63.6-87.0	65.8-90.8	56.0-77.2	62.7-83.8
Unknown, n (RP)	7,955 (39.6)	4,006 (52.8)	4,096 (32.8)	667 (40.3)	103 (32.9)	283 (61.0)	456 (72.4)
Country							
United States, n (RP)	11,397 (56.8)	2,318 (30.5)	7,355 (58.9)	853 (51.5)	255 (81.5)	54 (11.6)	466 (74.0)
Canada, n (RP)	370 (1.8)	39 (0.5)	507 (4.1)	19 (1.1)	5 (1.6)	2 (0.4)	0 (0.0)
Brazil, n (RP)	571 (2.8)	88 (1.2)	354 (2.8)	0 (0.0)	1 (0.3)	16 (3.4)	10 (1.6)
Venezuela, n (RP)	763 (3.8)	0 (0.0)	0 (0.0)	0 (0.0)	0 (0.0)	0 (0.0)	0 (0.0)
Japan, n (RP)	399 (2.0)	13 (0.2)	351 (2.8)	20 (1.2)	0 (0.0)	126 (27.2)	102 (16.2)
Denmark, n (RP)	86 (0.4)	128 (1.7)	21 (0.2)	4 (0.2)	0 (0.0)	0 (0.0)	0 (0.0)
France, n (RP)	795 (4.0)	310 (4.1)	356 (2.8)	166 (10.0)	0 (0.0)	46 (9.9)	0 (0.0)
Germany, n (RP)	316 (1.6)	271 (3.6)	176 (1.4)	31 (1.9)	1 (0.3)	22 (4.7)	0 (0.0)
Italy, n (RP)	344 (1.7)	190 (2.5)	108 (0.9)	20 (1.2)	7 (2.2)	9 (1.9)	0 (0.0)
Netherlands, n (RP)	349 (1.7)	204 (2.7)	216 (1.7)	46 (2.8)	0 (0.0)	12 (2.6)	3 (0.5)
Spain, n (RP)	220 (1.1)	80 (1.1)	41 (0.3)	9 (0.5)	2 (0.6)	9 (1.9)	10 (1.6)
United Kingdom, n (RP)	2,177 (10.8)	2,608 (34.3)	507 (4.1)	187 (11.3)	1 (0.3)	8 (1.7)	0 (0.0)
Australia, n (RP)	138 (0.7)	26 (0.3)	63 (0.5)	5 (0.3)	0 (0.0)	1 (0.2)	0 (0.0)
Others, n (RP)	962 (4.8)	461 (6.1)	510 (4.1)	31 (1.9)	4 (1.3)	66 (14.2)	35 (5.6)
Unknown, n (RP)	1,188 (5.9)	857 (11.3)	1,928 (15.4)	264 (16.0)	37 (11.8)	93 (20.0)	4 (0.6)

## Results

Adverse events

Table 2 summarizes the AE categories for each statin. Table 3 presents the ROR and aROR for AE categories, using atorvastatin as the reference. While aROR is generally preferred over ROR, the sample size for aROR calculations was limited due to numerous missing data in the FAERS database. Specifically, while all 43,223 cases were used for ROR calculations, only 30,430 cases were utilized for aROR calculations due to substantial missing data in (sex), (age), and (reporter_country) variables. Consequently, this study concentrated on statins exhibiting p < 0.0024 for both ROR (which uses all patient data but does not adjust for differences in patient background variables) and aROR (which adjusts for patient background variable differences but uses data from only a subset of patients due to missing data exclusion). This conservative approach has the benefit of reducing false positives by requiring significance in both ROR and aROR analyses. By focusing on statins that show significance in both measures, we can have greater confidence in the robustness of the identified associations. Compared to atorvastatin, both aROR and ROR for musculoskeletal disorders were significantly > 1 in simvastatin, lovastatin, and pitavastatin. Similar patterns of both significantly aROR > 1 and ROR > 1 were found for pain disorders in rosuvastatin; neurological disorders in simvastatin, rosuvastatin, and lovastatin; gastrointestinal disorders in simvastatin, rosuvastatin, pravastatin, fluvastatin, and pitavastatin; general fatigue disorders in simvastatin, rosuvastatin, and lovastatin; dermatological disorders in rosuvastatin; and hepatic disorders in fluvastatin. Conversely, metabolic disorders demonstrated significant aROR < 1 for simvastatin, rosuvastatin, pravastatin, lovastatin, and pitavastatin. The RPs in AE categories, excluding metabolic disorders, tended to be lower for other statins compared to atorvastatin. In the two AE categories considered for efficacy evaluation, direct treatment inefficacy indicators showed both significant ROR < 1 and aROR < 1 for simvastatin and pravastatin, while indirect treatment inefficacy indicators exhibited significant aROR < 1 for simvastatin and pravastatin. These findings suggest that atorvastatin may exhibit a relatively favorable safety profile compared to other statins in certain AE categories. However, it is important to note that these results also indicate the possibility of a lower efficacy profile for atorvastatin in comparison to other statins. This nuanced interpretation underscores the need for a comprehensive evaluation of both safety and efficacy when considering statin therapy options.

**Table 2:** The ROR and aROR For ROR and aROR, the reference for statin type, sex, and country were set to atorvastatin, female, and other countries, respectively. The sample sizes used for ROR were 43,223 for statin type; 41,090 for sex; 34,591 for age; and 38,852 for country. The sample size employed for aROR remained consistent at 30,430. aROR: adjusted reporting odds ratio; CI: confidence interval; ROR: reporting odds ratio

Items	Univariate analysis		Multivariate analysis	
	ROR (99.76%CI)	p-value	aROR (99.76%CI)	p-value
Musculoskeletal disorders				
Simvastatin	1.965 (1.805–2.139)	<0.0001	2.328 (2.097–2.583)	<0.0001
Rosuvastatin	1.014 (0.939–1.095)	0.5724	1.180 (1.076–1.294)	<0.0001
Pravastatin	1.063 (0.896–1.260)	0.2773	1.352 (1.101–1.661)	<0.0001
Lovastatin	1.708 (1.199–2.431)	<0.0001	1.735 (1.134–2.657)	0.0001
Fluvastatin	0.934 (0.678–1.289)	0.5219	1.200 (0.799–1.802)	0.1728
Pitavastatin	1.418 (1.097–1.833)	<0.0001	1.687 (1.186–2.400)	<0.0001
Male	1.223 (1.146–1.306)	<0.0001	1.256 (1.161–1.358)	<0.0001
Age	1.001 (0.998–1.003)	0.5671	1.002 (0.999–1.005)	0.0904
United States	1.168 (1.091–1.250)	<0.0001	1.386 (1.277–1.504)	<0.0001
Pain disorders				
Simvastatin	1.058 (0.952–1.176)	0.1043	1.276 (1.120–1.454)	<0.0001
Rosuvastatin	1.169 (1.071–1.276)	<0.0001	1.278 (1.150–1.420)	<0.0001
Pravastatin	1.075 (0.881–1.311)	0.2712	1.319 (1.041–1.672)	0.0004
Lovastatin	1.718 (1.163–2.537)	<0.0001	1.509 (0.940–2.420)	0.0082
Fluvastatin	0.711 (0.468–1.080)	0.0131	0.906 (0.524–1.568)	0.5846
Pitavastatin	1.595 (1.202–2.116)	<0.0001	1.404 (0.931–2.119)	0.0122
Male	0.893 (0.826–0.965)	<0.0001	0.993 (0.905–1.089)	0.8109
Age	0.998 (0.995–1.002)	0.1307	0.998 (0.995–1.002)	0.1951
United States	1.714 (1.576–1.864)	<0.0001	1.782 (1.614–1.967)	<0.0001
Neurological disorders				
Simvastatin	1.239 (1.120–1.372)	<0.0001	1.619 (1.433–1.828)	<0.0001
Rosuvastatin	1.146 (1.050–1.251)	<0.0001	1.235 (1.112–1.373)	<0.0001
Pravastatin	1.210 (1.000–1.465)	0.0024	1.519 (1.210–1.905)	<0.0001
Lovastatin	2.049 (1.409–2.980)	<0.0001	2.312 (1.490–3.587)	<0.0001
Fluvastatin	0.772 (0.516–1.155)	0.0508	0.874 (0.515–1.483)	0.4382
Pitavastatin	1.332 (0.993–1.786)	0.0030	1.402 (0.932–2.108)	0.0119
Male	0.878 (0.813–0.948)	<0.0001	0.897 (0.819–0.983)	0.0003
Age	0.995 (0.991–0.998)	<0.0001	0.994 (0.991–0.998)	<0.0001
United States	1.293 (1.194–1.401)	<0.0001	1.413 (1.285–1.554)	<0.0001
Gastrointestinal disorders				
Simvastatin	1.283 (1.124–1.464)	<0.0001	1.497 (1.279–1.752)	<0.0001
Rosuvastatin	1.582 (1.420–1.763)	<0.0001	1.654 (1.453–1.882)	<0.0001
Pravastatin	1.490 (1.180–1.882)	<0.0001	1.598 (1.204–2.122)	<0.0001
Lovastatin	1.243 (0.717–2.155)	0.2291	1.211 (0.622–2.356)	0.3825
Fluvastatin	1.814 (1.220–2.696)	<0.0001	1.854 (1.115–3.082)	0.0002
Pitavastatin	1.819 (1.292–2.561)	<0.0001	2.240 (1.437–3.492)	<0.0001
Male	0.676 (0.612–0.747)	<0.0001	0.697 (0.621–0.783)	<0.0001
Age	0.999 (0.995–1.004)	0.6496	0.997 (0.993–1.002)	0.0805
United States	1.104 (0.999–1.220)	0.0028	1.107 (0.984–1.245)	0.0090
General fatigue disorders				
Simvastatin	1.572 (1.408–1.756)	<0.0001	1.880 (1.648–2.145)	<0.0001
Rosuvastatin	1.213 (1.098–1.340)	<0.0001	1.295 (1.148–1.460)	<0.0001
Pravastatin	1.065 (0.848–1.338)	0.3982	1.249 (0.951–1.640)	0.0130
Lovastatin	1.690 (1.093–2.613)	0.0003	2.209 (1.352–3.609)	<0.0001
Fluvastatin	1.490 (1.026–2.166)	0.0012	1.603 (0.995–2.582)	0.0027
Pitavastatin	1.200 (0.849–1.695)	0.1094	1.470 (0.932–2.319)	0.0102
Male	1.007 (0.925–1.097)	0.7960	1.016 (0.918–1.124)	0.6382
Age	0.996 (0.992–0.999)	0.0002	0.995 (0.991–0.999)	0.0001
United States	0.967 (0.884–1.056)	0.2450	1.088 (0.980–1.209)	0.0145
Dermatological disorders				
Simvastatin	1.110 (0.946–1.302)	0.0474	1.310 (1.084–1.582)	<0.0001
Rosuvastatin	1.304 (1.145–1.486)	<0.0001	1.392 (1.191–1.628)	<0.0001
Pravastatin	1.667 (1.287–2.158)	<0.0001	1.380 (0.977–1.950)	0.0047
Lovastatin	1.125 (0.579–2.185)	0.5897	1.085 (0.477–2.467)	0.7638
Fluvastatin	0.573 (0.274–1.194)	0.0212	0.569 (0.222–1.460)	0.0693
Pitavastatin	1.438 (0.938–2.206)	0.0098	1.302 (0.701–2.419)	0.1958
Male	0.658 (0.583–0.743)	<0.0001	0.705 (0.612–0.812)	<0.0001
Age	1.000 (0.995–1.005)	0.9755	0.999 (0.994–1.005)	0.7478
United States	1.097 (0.973–1.237)	0.0190	1.019 (0.884–1.174)	0.6892
Hepatic disorders				
Simvastatin	1.120 (0.945–1.327)	0.0421	1.008 (0.823–1.235)	0.9039
Rosuvastatin	0.707 (0.600–0.834)	<0.0001	0.848 (0.696–1.032)	0.0108
Pravastatin	0.920 (0.651–1.299)	0.4628	1.070 (0.709–1.614)	0.6194
Lovastatin	1.360 (0.709–2.611)	0.1515	2.286 (1.086–4.812)	0.0007
Fluvastatin	4.386 (3.067–6.272)	<0.0001	3.721 (2.397–5.777)	<0.0001
Pitavastatin	1.140 (0.692–1.879)	0.4244	2.078 (1.188–3.634)	0.0001
Male	1.050 (0.922–1.197)	0.2537	0.960 (0.822–1.121)	0.4222
Age	1.002 (0.996–1.008)	0.2863	1.002 (0.996–1.008)	0.2979
United States	0.450 (0.391–0.518)	<0.0001	0.445 (0.377–0.524)	<0.0001
Metabolic disorders				
Simvastatin	0.178 (0.146–0.217)	<0.0001	0.212 (0.166–0.272)	<0.0001
Rosuvastatin	0.432 (0.385–0.485)	<0.0001	0.347 (0.302–0.399)	<0.0001
Pravastatin	0.213 (0.146–0.311)	<0.0001	0.175 (0.109–0.280)	<0.0001
Lovastatin	0.426 (0.225–0.804)	<0.0001	0.209 (0.092–0.473)	<0.0001
Fluvastatin	0.291 (0.157–0.539)	<0.0001	0.704 (0.337–1.472)	0.1482
Pitavastatin	0.306 (0.182–0.514)	<0.0001	0.270 (0.131–0.555)	<0.0001
Male	0.365 (0.327–0.408)	<0.0001	0.321 (0.281–0.368)	<0.0001
Age	0.969 (0.965–0.973)	<0.0001	0.964 (0.959–0.968)	<0.0001
United States	4.605 (4.044–5.244)	<0.0001	4.845 (4.163–5.638)	<0.0001
Direct treatment inefficacy indicators				
Simvastatin	0.393 (0.321–0.482)	<0.0001	0.540 (0.417–0.701)	<0.0001
Rosuvastatin	0.742 (0.649–0.849)	<0.0001	0.881 (0.747–1.040)	0.0204
Pravastatin	0.499 (0.345–0.722)	<0.0001	0.393 (0.226–0.685)	<0.0001
Lovastatin	0.689 (0.334–1.421)	0.1179	0.718 (0.308–1.674)	0.2342
Fluvastatin	0.430 (0.207–0.897)	0.0005	0.346 (0.087–1.372)	0.0193
Pitavastatin	0.368 (0.187–0.725)	<0.0001	0.364 (0.130–1.025)	0.0030
Male	0.902 (0.799–1.017)	0.0091	1.025 (0.880–1.194)	0.6224
Age	0.998 (0.992–1.003)	0.2345	1.001 (0.995–1.007)	0.6699
United States	2.855 (2.464–3.309)	<0.0001	2.383 (1.999–2.840)	<0.0001
Indirect treatment inefficacy indicators				
Simvastatin	0.616 (0.512–0.740)	<0.0001	0.517 (0.412–0.649)	<0.0001
Rosuvastatin	0.790 (0.686–0.910)	<0.0001	0.896 (0.759–1.058)	0.0453
Pravastatin	0.262 (0.154–0.446)	<0.0001	0.266 (0.139–0.508)	<0.0001
Lovastatin	0.498 (0.203–1.222)	0.0182	0.735 (0.284–1.898)	0.3239
Fluvastatin	1.018 (0.593–1.748)	0.9185	0.638 (0.289–1.408)	0.0848
Pitavastatin	0.409 (0.204–0.820)	0.0001	0.433 (0.170–1.107)	0.0068
Male	1.623 (1.434–1.837)	<0.0001	1.641 (1.418–1.900)	<0.0001
Age	1.012 (1.006–1.018)	<0.0001	1.012 (1.006–1.019)	<0.0001
United States	0.855 (0.752–0.972)	0.0002	0.801 (0.690–0.931)	<0.0001

**Table 3:** Summary of adverse event category Data are summarized as frequency (reporting proportion). If multiple adverse events are reported in a patient, each adverse event is counted. Each patient is counted only once per adverse event category, regardless of the number of preferred terms experienced within that category.

Adverse event category	Atorvastatin	Simvastatin	Rosuvastatin	Pravastatin	Lovastatin	Fluvastatin	Pitavastatin
	N = 20,075	N = 7,593	N = 12,493	N = 1,655	N = 313	N = 464	N = 630
Musculoskeletal disorders	5,680 (28.3)	3,316 (43.7)	3,571 (28.6)	489 (29.5)	126 (40.3)	125 (26.9)	226 (35.9)
Pain disorders	3,580 (17.8)	1,418 (18.7)	2,528 (20.2)	313 (18.9)	85 (27.2)	62 (13.4)	162 (25.7)
Neurological disorders	3,653 (18.2)	1,641 (21.6)	2,538 (20.3)	351 (21.2)	98 (31.3)	68 (14.7)	144 (22.9)
Gastrointestinal disorders	1,846 (9.2)	873 (11.5)	1,725 (13.8)	217 (13.1)	35 (11.2)	72 (15.5)	98 (15.6)
General fatigue disorders	2,560 (12.8)	1,419 (18.7)	1,881 (15.1)	223 (13.5)	62 (19.8)	83 (17.9)	94 (14.9)
Dermatological disorders	1,322 (6.6)	551 (7.3)	1,052 (8.4)	174 (10.5)	23 (7.3)	18 (3.9)	58 (9.2)
Hepatic disorders	1,155 (5.8)	486 (6.4)	517 (4.1)	88 (5.3)	24 (7.7)	98 (21.1)	41 (6.5)
Metabolic disorders	3,400 (16.9)	266 (3.5)	1,012 (8.1)	69 (4.2)	25 (8.0)	26 (5.6)	37 (5.9)
Direct treatment inefficacy indicators	1,721 (8.6)	270 (3.6)	813 (6.5)	74 (4.5)	19 (6.1)	18 (3.9)	21 (3.3)
Indirect treatment inefficacy indicators	1,489 (7.4)	357 (4.7)	744 (6.0)	34 (2.1)	12 (3.8)	35 (7.5)	20 (3.2)

Appendix B summarizes the results of both ROR and aROR using each of the other six statins sequentially as the reference, presented as one of three statistical test outcomes: both significantly ROR < 1 and aROR < 1, both significantly ROR > 1 and aROR > 1, and at least one of ROR or aROR is not significant. Given that both ROR and aROR maintain their fundamental odds ratio properties [15], the detailed results for ROR and aROR using different statins as the reference can be readily derived from Table 3 [16]. The detailed calculation procedure is provided in Appendix C. No statin demonstrated consistently low RPs across all AE categories. Each statin exhibited a mix of higher and lower RPs depending on the AE category.

## Discussion

This study's strengths lie in its focused approach and stringent inclusion criteria. By exclusively analyzing patients using statins for hypercholesterolemia management, the research maintains a high degree of specificity and relevance. Moreover, the study's scope was further refined by limiting the analysis to AEs in which statins were identified as the primary suspect drugs. This methodological rigor enhances the reliability of the findings and minimizes potential confounding factors, thereby providing a more accurate assessment of statin-related AEs in the context of hypercholesterolemia treatment. Furthermore, our comprehensive comparison of all seven primary statins, coupled with the application of the Bonferroni method for significance level adjustment, ensures statistical robustness and mitigates the risk of type I errors. This approach bolsters the credibility of our findings, offering a solid foundation for informed clinical decision-making in statin therapy for hypercholesterolemia management.

The evolution of statins is characterized by two distinct expansions: the diversification of statin types and the broadening of their therapeutic applications. Initially developed for hypercholesterolemia, statins have grown into a family of seven primary drugs [2]. Concurrently, their efficacy was found to extend beyond cholesterol reduction to preventing stroke [17] and managing diabetes mellitus [18]. This dual expansion necessitates comprehensive comparisons to guide clinical decision-making in an increasingly complex therapeutic landscape.

Comparing all seven statins in a single clinical trial would necessitate an exceptionally large sample size, which may be difficult to achieve. Consequently, researchers have turned to alternative methodologies, such as utilizing the FAERS database, to conduct comparative studies of statins and overcome these limitations. Because statins have different AE trends depending on the type of disease [4], studies using the FAERS database needed to limit the reasons for statin administration. However, while previous studies have utilized FAERS data to evaluate AEs across seven statins, many studies fail to specify whether they focused on a particular indication for statin therapy [10,11]. This ambiguity suggests that data from all indications may have been pooled, potentially compromising the control of confounding factors. Therefore, there is a need for transparent methodologies to avoid misleading conclusions regarding the efficacy and safety of statins [19]. In light of these concerns, this study was designed to address the methodological limitations of prior research by leveraging the FAERS database to rigorously compare the safety profiles of statins across specific indications, thereby ensuring more robust control of confounding factors.

All seven statins demonstrated high RP > 25 for musculoskeletal disorders. These findings align with previous studies that have frequently documented musculoskeletal disorders associated with statin therapy [8]. The consistency of these observations across different statin types suggests that these musculoskeletal disorder AEs are not specific to individual statins but rather a characteristic of the statin class as a whole. Atorvastatin exhibited the highest RP for type 2 diabetes mellitus, followed closely by rosuvastatin, whereas the other five statins showed low RPs. This supports previous studies suggesting that more potent statins are associated with a higher risk of diabetes development [20,21]. The RPs for gastrointestinal disorders ranged from 9 to 16 across all statins, while those for dermatological disorders ranged from 3 to 11. Compared to other AE categories, these two categories showed relatively small variability among different statins. This suggests that these AEs may not be attributable to individual statin-specific reactions but rather reflect a class-wide effect. AEs caused by statins are generally considered mild [22]; however, they may pose secondary challenges by hindering regular medication adherence. For instance, symptoms such as nausea, fatigue, dizziness, and malaise may lead patients to skip doses or discontinue treatment [23], ultimately reducing the intended therapeutic efficacy.

The higher RP of female patients observed for most statins in our study aligns with previous research showing higher RPs of AE reporting among females [24]. This pattern may be attributed to multiple factors, including pharmacokinetic differences [25], estrogen interactions [26], and genetic polymorphisms, such as SLCO1B1 variants [27]. However, the strength of evidence varies among these explanations. Pharmacokinetic differences between sexes are well-documented and provide strong evidence for increased susceptibility to statin-related AEs in females. Women generally exhibit higher systemic exposure to statins due to differences in body composition, including lower muscle mass and higher fat percentages, which influence drug distribution and metabolism. Additionally, faster metabolism of CYP3A4 substrates in women may contribute to altered pharmacokinetics, potentially exacerbating adverse effects. The role of SLCO1B1 polymorphisms, particularly the rs4149056 variant, has gained increasing attention as a genetic factor influencing statin-related AEs. Studies suggest that this polymorphism leads to elevated plasma levels of statins and an increased risk of muscle-related side effects, particularly in women. Evidence also indicates that women with this polymorphism are less likely to achieve LDL-C targets, suggesting a sex-specific impact on both efficacy and safety profiles. While promising, further research is needed to fully elucidate these interactions and their clinical implications. In contrast, the evidence for direct interactions between statins and estrogen metabolism remains less robust. Although competitive inhibition at CYP enzymes is theoretically plausible due to shared metabolic pathways, clinical studies directly linking estrogen interactions with increased AE risks are limited. This area warrants further investigation to determine its significance in real-world settings. Finally, reporting biases and healthcare utilization differences may contribute to the observed gender disparities but are challenging to quantify and interpret due to variability across healthcare systems and cultural contexts.

Despite the well-established benefits of statin therapy, our results suggest that atorvastatin had higher RPs of two efficacy categories (direct and indirect treatment inefficacy indicators) than other statins. These findings align with previous research utilizing large-scale databases. A study employing the General Electric Centricity Electronic Medical Record and Humana Medicare databases reported that a significant number of high-risk cardiovascular disease patients failed to achieve guideline-recommended LDL-C levels with atorvastatin monotherapy [28]. The higher RP of treatment inefficacy for atorvastatin may be attributed to various factors, including its unique pharmacokinetic properties. Atorvastatin has a longer half-life (approximately 14 hours) compared to most other statins (one to three hours), with its active metabolites extending the effect to 20-30 hours. This prolonged action, while generally beneficial for maintaining lipid-lowering effects, may also lead to extended systemic exposure, potentially increasing the likelihood of AEs. Furthermore, atorvastatin's higher lipophilicity allows for greater penetration into peripheral tissues. While this property enhances its hepatic uptake and cholesterol-lowering efficacy, it may also contribute to a higher incidence of AEs in non-hepatic tissues. These AEs could potentially lead to treatment discontinuation or dose reduction, indirectly affecting treatment efficacy. It is important to note that the relationship between these pharmacokinetic factors and treatment inefficacy is likely indirect. Rather than directly causing treatment resistance, these properties may influence AE trends, patient adherence, and dosing strategies, which in turn affect overall treatment efficacy. Additionally, atorvastatin is often prescribed at higher doses or to patients with more severe hypercholesterolemia, which could influence both efficacy and AE reporting. Patients on higher doses or with more severe disease may be more closely monitored, potentially leading to increased reporting of both inefficacy and AEs. The consistency between our findings, derived from a large-scale FAERS database, and those of the aforementioned study underscores the importance of utilizing extensive sample sizes in statin research. This approach provides a more comprehensive understanding of real-world drug effectiveness and safety profiles. The discrepancies observed between clinical trial outcomes and actual patient experiences highlight the complex nature of statin efficacy in diverse patient populations.

This study revealed that fluvastatin had a notably high RP > 20 for hepatic disorders, while the other six statins showed RPs < 10. This finding aligns with previous research, which reported a signal for hepatic reactions associated with fluvastatin based on spontaneous reporting in Italy [29]. The higher risk of hepatic disorders with fluvastatin may be attributed to its unique pharmacokinetic properties and metabolism [30]. Fluvastatin is primarily metabolized by the CYP2C9 enzyme in the liver, unlike other statins that are predominantly metabolized by CYP3A4. This distinct metabolic pathway, coupled with fluvastatin's short elimination half-life and high liver extraction, results in higher liver concentrations, potentially increasing the risk of hepatocellular injury, particularly in patients with pre-existing liver conditions. The mechanism behind fluvastatin's increased hepatotoxicity risk may involve its effects on organic anion transporter organic anion transporting polypeptide 1B1 and the reduced metabolic activity of CYP2C9 variants.

The significantly higher RP of metabolic disorders with atorvastatin compared to other statins may be due to its pharmacokinetic properties, including longer half-life and higher lipophilicity, as well as its frequent use at higher doses. However, reporting biases should also be considered when interpreting these findings. The observed variations in AE associations across statins likely reflect differences in their pharmacokinetic properties. For instance, lipophilic statins such as simvastatin may have greater tissue penetration, potentially explaining the higher RP of musculoskeletal disorders. Conversely, hydrophilic statins such as pravastatin may have lower tissue distribution outside the liver, possibly contributing to different AE profiles.

This study observed that some countries, such as Venezuela, reported cases only for specific statins. This pattern could be attributed to several factors. Differences in drug availability and approval status across countries play a significant role. Additionally, variations in prescribing patterns and clinical guidelines contribute to these disparities. Potential reporting biases or differences in pharmacovigilance systems among nations may also influence the observed patterns. Furthermore, the market penetration of different statins in various regions can impact the reporting trends. These country-specific patterns underscore the importance of considering geographical variations in drug use and AE reporting when interpreting pharmacovigilance data. Such variations can provide valuable insights into regional healthcare practices and potential areas for further investigation in drug safety monitoring. While a detailed country-by-country analysis was beyond the scope of this study, our findings provide a valuable global overview of AE reporting patterns for statins. Future research could explore these geographical variations in more depth, potentially yielding insights into regional healthcare practices and areas for targeted pharmacovigilance efforts.

This study has several limitations due to the use of the FAERS database. First, we were unable to determine the incidence of AEs since the FAERS database only reports occurrences. Second, the data are prone to notoriety bias. Third, there are numerous incomplete or missing entries. Fourth, patients' medical histories were largely unknown, except for hypercholesterolemia. Finally, our study was limited in its ability to account for statin dosage, which can significantly impact both efficacy and AE profiles. Furthermore, patients experiencing AEs may have different underlying health conditions or risk factors that influence their response to statin therapy. Despite these limitations, the FAERS database provides the benefit of a vast number of reports from around the globe, allowing us to maintain a sufficient sample size for comparison across different seven statins, even after applying strict inclusion and exclusion criteria to minimize potential confounding factors. Therefore, our findings were considered hypothesis-generating rather than definitive evidence. To bridge this gap, future studies should aim to validate these results against data from clinical registries, electronic health records, or prospective observational studies. Such validation would provide a more comprehensive understanding of the safety profile of statins in real-world settings and help clinicians make more informed decisions about statin therapy for their patients.

## Conclusions

This comprehensive analysis of AE profiles among seven statins for hypercholesterolemia management reveals notable variations, particularly in musculoskeletal disorders, metabolic effects, and treatment efficacy indicators. For instance, we observed higher RP of musculoskeletal disorders with simvastatin and rosuvastatin compared to atorvastatin, while atorvastatin showed higher RP of metabolic disorders. These findings generate hypotheses about potential differences in statin safety and efficacy profiles that warrant further investigation. Our results suggest the possibility of tailoring statin selection based on individual patient risk factors. For example, patients with preexisting muscle disorders might benefit from statins with lower RPs of musculoskeletal AEs, such as atorvastatin. For patients with diabetes risk factors, closer monitoring may be warranted when prescribing atorvastatin, which showed the highest RP of metabolic disorders compared to other statins in our analysis. However, it is crucial to emphasize that these hypotheses require validation through more rigorous study designs. Given the limitations of the FAERS database, including potential reporting biases and lack of incidence data, our findings should be interpreted as generating hypotheses for further research rather than providing definitive clinical recommendations. To validate these findings in real-world clinical settings and establish causal relationships, we propose a stepwise approach to future research. As an initial step, well-designed observational studies, particularly propensity-matched cohort studies, could provide valuable insights while being more feasible than immediate large-scale clinical trials. These studies can help control for confounding factors and offer a more robust assessment of the comparative safety and efficacy of different statins in specific patient populations. Following these observational studies, large-scale registry studies and randomized controlled trials would be crucial for definitively establishing causal relationships and developing evidence-based guidelines for personalized statin selection. This research trajectory will ultimately enhance the quality and safety of cardiovascular care by providing a stronger evidence base for tailored statin therapy.

## Appendices

Appendix A

**Table 4:** Comparative characteristics of seven statins CYP3A4: cytochrome P450 family 3 subfamily A member 4; CYP2C9: cytochrome P450 family 2 subfamily C member 9; UGT1A3: uridine diphosphate-glucuronosyltransferase family 1 member A3; UGT2B7: uridine diphosphate-glucuronosyltransferase family 2 member B7

Statin	Lipophilicity	Metabolism	Half-life (hours)	Low-density lipoprotein cholesterol reduction (%)	Main excretion route
Atorvastatin	Lipophilic	CYP3A4	14	38–54	Biliary
Simvastatin	Lipophilic	CYP3A4	2–3	28–41	Biliary
Rosuvastatin	Hydrophilic	CYP2C9 (minimal)	19	45–63	Biliary
Pravastatin	Hydrophilic	Sulfation	1–3	20–30	Renal and Biliary
Lovastatin	Lipophilic	CYP3A4	2–3	29–40	Biliary
Fluvastatin	Lipophilic	CYP2C9	0.5–2.3	17–23	Biliary
Pitavastatin	Lipophilic	UGT1A3, UGT2B7	11	32–43	Biliary

Appendix B

**Table 5:** Cross-tabulation of adverse event categories and statins with significant adjusted reporting odds ratio < 1: Indicates both ROR and aROR are significantly < 1 compared to the reference statin; > 1: Indicates both ROR and aROR are significant> 1 compared to the reference statin; N.S.: At least one of ROR or aROR is not significant compared to the reference statin, aROR: adjusted reporting odds ratio; ROR: reporting odds ratio

Adverse event category	Atorvastatin	Simvastatin	Rosuvastatin	Pravastatin	Lovastatin	Fluvastatin	Pitavastatin
Musculoskeletal disorders	< 1	Reference	< 1	< 1	N.S.	< 1	N.S.
Pain disorders	N.S.	Reference	N.S.	N.S.	N.S.	N.S.	N.S.
Neurological disorders	< 1	Reference	N.S.	N.S.	N.S.	< 1	N.S.
Gastrointestinal disorders	< 1	Reference	N.S.	N.S.	N.S.	N.S.	N.S.
General fatigue disorders	< 1	Reference	< 1	< 1	N.S.	N.S.	N.S.
Dermatological disorders	N.S.	Reference	N.S.	N.S.	N.S.	N.S.	N.S.
Hepatic disorders	N.S.	Reference	N.S.	N.S.	N.S.	> 1	N.S.
Metabolic disorders	> 1	Reference	> 1	N.S.	N.S.	N.S.	N.S.
Direct treatment inefficacy indicators	> 1	Reference	> 1	N.S.	N.S.	N.S.	N.S.
Indirect treatment inefficacy indicators	> 1	Reference	> 1	N.S.	N.S.	N.S.	N.S.
Musculoskeletal disorders	N.S.	> 1	Reference	N.S.	N.S.	N.S.	> 1
Pain disorders	< 1	N.S.	Reference	N.S.	N.S.	N.S.	N.S.
Neurological disorders	< 1	N.S.	Reference	N.S.	> 1	N.S.	N.S.
Gastrointestinal disorders	< 1	N.S.	Reference	N.S.	N.S.	N.S.	N.S.
General fatigue disorders	< 1	> 1	Reference	N.S.	N.S.	N.S.	N.S.
Dermatological disorders	< 1	N.S.	Reference	N.S.	N.S.	N.S.	N.S.
Hepatic disorders	N.S.	N.S.	Reference	N.S.	N.S.	> 1	N.S.
Metabolic disorders	> 1	< 1	Reference	< 1	N.S.	N.S.	N.S.
Direct treatment inefficacy indicators	N.S.	< 1	Reference	< 1	N.S.	N.S.	N.S.
Indirect treatment inefficacy indicators	N.S.	< 1	Reference	< 1	N.S.	N.S.	N.S.
Musculoskeletal disorders	N.S.	> 1	N.S.	Reference	N.S.	N.S.	N.S.
Pain disorders	N.S.	N.S.	N.S.	Reference	N.S.	N.S.	N.S.
Neurological disorders	N.S.	N.S.	N.S.	Reference	N.S.	N.S.	N.S.
Gastrointestinal disorders	< 1	N.S.	N.S.	Reference	N.S.	N.S.	N.S.
General fatigue disorders	N.S.	> 1	N.S.	Reference	N.S.	N.S.	N.S.
Dermatological disorders	N.S.	N.S.	N.S.	Reference	N.S.	N.S.	N.S.
Hepatic disorders	N.S.	N.S.	N.S.	Reference	N.S.	> 1	N.S.
Metabolic disorders	> 1	N.S.	> 1	Reference	N.S.	N.S.	N.S.
Direct treatment inefficacy indicators	> 1	N.S.	> 1	Reference	N.S.	N.S.	N.S.
Indirect treatment inefficacy indicators	> 1	N.S.	> 1	Reference	N.S.	N.S.	N.S.
Musculoskeletal disorders	< 1	N.S.	N.S.	N.S.	Reference	N.S.	N.S.
Pain disorders	N.S.	N.S.	N.S.	N.S.	Reference	N.S.	N.S.
Neurological disorders	< 1	N.S.	< 1	N.S.	Reference	< 1	N.S.
Gastrointestinal disorders	N.S.	N.S.	N.S.	N.S.	Reference	N.S.	N.S.
General fatigue disorders	< 1	N.S.	N.S.	N.S.	Reference	N.S.	N.S.
Dermatological disorders	N.S.	N.S.	N.S.	N.S.	Reference	N.S.	N.S.
Hepatic disorders	N.S.	N.S.	N.S.	N.S.	Reference	N.S.	N.S.
Metabolic disorders	> 1	N.S.	N.S.	N.S.	Reference	N.S.	N.S.
Direct treatment inefficacy indicators	N.S.	N.S.	N.S.	N.S.	Reference	N.S.	N.S.
Indirect treatment inefficacy indicators	N.S.	N.S.	N.S.	N.S.	Reference	N.S.	N.S.
Musculoskeletal disorders	N.S.	> 1	N.S.	N.S.	N.S.	Reference	N.S.
Pain disorders	N.S.	N.S.	N.S.	N.S.	N.S.	Reference	N.S.
Neurological disorders	N.S.	> 1	N.S.	N.S.	> 1	Reference	N.S.
Gastrointestinal disorders	< 1	N.S.	N.S.	N.S.	N.S.	Reference	N.S.
General fatigue disorders	N.S.	N.S.	N.S.	N.S.	N.S.	Reference	N.S.
Dermatological disorders	N.S.	N.S.	N.S.	N.S.	N.S.	Reference	N.S.
Hepatic disorders	< 1	< 1	< 1	< 1	N.S.	Reference	N.S.
Metabolic disorders	N.S.	N.S.	N.S.	N.S.	N.S.	Reference	N.S.
Direct treatment inefficacy indicators	N.S.	N.S.	N.S.	N.S.	N.S.	Reference	N.S.
Indirect treatment inefficacy indicators	N.S.	N.S.	N.S.	N.S.	N.S.	Reference	N.S.
Musculoskeletal disorders	< 1	N.S.	< 1	N.S.	N.S.	N.S.	Reference
Pain disorders	N.S.	N.S.	N.S.	N.S.	N.S.	N.S.	Reference
Neurological disorders	N.S.	N.S.	N.S.	N.S.	N.S.	N.S.	Reference
Gastrointestinal disorders	< 1	N.S.	N.S.	N.S.	N.S.	N.S.	Reference
General fatigue disorders	N.S.	N.S.	N.S.	N.S.	N.S.	N.S.	Reference
Dermatological disorders	N.S.	N.S.	N.S.	N.S.	N.S.	N.S.	Reference
Hepatic disorders	N.S.	N.S.	N.S.	N.S.	N.S.	N.S.	Reference
Metabolic disorders	> 1	N.S.	N.S.	N.S.	N.S.	N.S.	Reference
Direct treatment inefficacy indicators	N.S.	N.S.	N.S.	N.S.	N.S.	N.S.	Reference
Indirect treatment inefficacy indicators	N.S.	N.S.	N.S.	N.S.	N.S.	N.S.	Reference

Appendix C

For all calculations, intermediate steps were carried out to five decimal places to maintain precision. Final results are presented rounded to three decimal places for clarity and consistency with the main text. This example demonstrates the process of calculating the ROR and aROR for rosuvastatin using simvastatin as the reference. The calculations are based on the ROR and aROR values for musculoskeletal disorders presented in Table 3. The ROR (99.76% CI) for simvastatin is 1.965 (1.805-2.139), while that for rosuvastatin is 1.014 (0.939-1.095). Similarly, the aROR (99.76% CI) for simvastatin is 2.328 (2.097-2.583), while that for rosuvastatin is 1.180 (1.076-1.294). To compute the ROR of rosuvastatin with simvastatin as the reference, the ROR of rosuvastatin is divided by that of simvastatin: 1.014 / 1.965 = 0.51603. The critical value for the upper tail of the standard normal distribution at (1 - 0.05 / (21 × 2)) is 3.03807. To determine the 99.76% CI for this new ROR, we first calculate the standard errors (SEs) for simvastatin and rosuvastatin: SE_s = (log(99.76% CI upper) - log(99.76% CI lower)) / (2 × 3.03807) = (log(2.139) - log(1.805)) / (2 × 3.03807) = 0.02794 for simvastatin and SE_r = (log(1.095) - log(0.939)) / (2 × 3.03807) = 0.02529 for rosuvastatin. The SE of the new ROR is calculated by taking the square root of the sum of the squares of SE_s and SE_r: SE_new = sqrt(SE_s^2 + SE_r^2) = sqrt(0.02794^2 + 0.02529^2) = 0.03769. The 99.76% CI for the new ROR is computed as follows: exp(log(0.51603) ± 3.03807 × 0.03769). This results in a lower bound of 0.46020 and an upper bound of 0.57863, yielding an ROR (99.76% CI) of 0.516 (99.76% CI: 0.460-0.579). The calculation of SEs uses an approximation based on confidence interval widths rather than raw data or sample variances, which may introduce minor imprecision in cases with small sample sizes or wide confidence intervals for RORs [16]. However, this approximation is generally reliable when N > 100. In this study, even lovastatin, which had the fewest reports, had an N > 300, making this approximation acceptable. To calculate the p-value, a z-test is performed: z = log(ROR) / SE_new = log(0.51603) / 0.03769 = -17.5. The corresponding two-tailed p-value can be obtained using a standard normal distribution table or statistical software. Using a similar procedure, the aROR of rosuvastatin with simvastatin as the reference is calculated as follows: 1.180 / 2.328 = 0.50687. The SEs for simvastatin and rosuvastatin are as follows: SE_s = (log(2.583) - log(2.097)) / (2 × 3.03807) = 0.03431 for simvastatin and SE_r = (log(1.294) - log(1.076)) / (2 × 3.03807) = 0.03036. The SE of the new aROR is as follows: sqrt(0.03431^2 + 0.03036^2) = 0.04581. The corresponding 99.76% CI is calculated as follows: exp(log(0.50687) ± 3.03807 × 0.04581). This yields a lower bound of 0.44102 and an upper bound of 0.58256, resulting in an aROR (99.76% CI) of 0.507 (99.76% CI: 0.441-0.583). To calculate the p-value, a z-test is performed: z = log(0.50687) / 0.04581 = -14.8. The corresponding two-tailed p-value can be obtained using a standard normal distribution table or statistical software.

To reproduce these calculations, we provide the following R code, which readers can easily adapt for other examples by modifying the first four lines.

ROR_s<-1.965; ROR_CIl_s<-1.805; ROR_CIu_s<-2.139 ROR_r<-1.014; ROR_CIl_r<-0.939; ROR_CIu_r<-1.095 aROR_s<-2.328; aROR_CIl_s<-2.097; aROR_CIu_s<-2.583 aROR_r<-1.180; aROR_CIl_r<-1.076; aROR_CIu_r<-1.294 ROR_SE_s<-(log(ROR_CIu_s)-log(ROR_CIl_s))/(2*3.03807) ROR_SE_r<-(log(ROR_CIu_r)-log(ROR_CIl_r))/(2*3.03807) ROR_new<-ROR_r/ROR_s ROR_SE_new<-sqrt(ROR_SE_s^2+ROR_SE_r^2) ROR_CI_new<-exp(log(ROR_new)+c(-1,1)*3.03807*ROR_SE_new) ROR_p_value<-2*(1-pnorm(abs(log(ROR_new)/ROR_SE_new))) result_ROR<-c(ROR_new,ROR_CI_new,ROR_p_value) names(result_ROR)<-c("aROR","99.76%CI lower","99.76%CI upper","p-value") aROR_SE_s<-(log(aROR_CIu_s)-log(aROR_CIl_s))/(2*3.03807) aROR_SE_r<-(log(aROR_CIu_r)-log(aROR_CIl_r))/(2*3.03807) aROR_new<-aROR_r/aROR_s aROR_SE_new<-sqrt(aROR_SE_s^2+aROR_SE_r^2) aROR_CI_new<-exp(log(aROR_new)+c(-1,1)*3.03807*aROR_SE_new) aROR_p_value<-2*(1-pnorm(abs(log(aROR_new)/aROR_SE_new))) result_aROR<-c(aROR_new,aROR_CI_new,aROR_p_value) names(result_aROR)<-c("aROR","99.76%CI lower","99.76%CI upper","p-value") result_ROR result_aROR

## References

[1] Dushenko N, Vysochyn M (2024). Examination of lipoprotein and lipid levels of adult patients with atherosclerosis, myocardial infarction, and stroke: a narrative review. Cureus.

[2] Endo A (2010). A historical perspective on the discovery of statins. Proc Jpn Acad Ser B Phys Biol Sci.

[3] Istvan ES, Deisenhofer J (2001). Structural mechanism for statin inhibition of HMG-CoA reductase. Science.

[4] Mach F, Baigent C, Catapano AL (2020). 2019 ESC/EAS guidelines for the management of dyslipidaemias: lipid modification to reduce cardiovascular risk. Eur Heart J.

[5] Schachter M (2005). Chemical, pharmacokinetic and pharmacodynamic properties of statins: an update. Fundam Clin Pharmacol.

[6] Iwaki Y, Lee W, Sugiyama Y (2019). Comparative and quantitative assessment on statin efficacy and safety: insights into inter-statin and inter-individual variability via dose- and exposure-response relationships. Expert Opin Drug Metab Toxicol.

[7] Collins R, Reith C, Emberson J (2016). Interpretation of the evidence for the efficacy and safety of statin therapy. Lancet.

[8] Hussain A, Kaler J, Ray SD (2023). The benefits outweigh the risks of treating hypercholesterolemia: the statin dilemma. Cureus.

[9] (2025). FDA Adverse Event Reporting System (FAERS) quarterly data extract files. https://fis.fda.gov/extensions/FPD-QDE-FAERS/FPD-QDE-FAERS.html.

[10] Hoffman KB, Kraus C, Dimbil M, Golomb BA (2012). A survey of the FDA's AERS database regarding muscle and tendon adverse events linked to the statin drug class. PLoS One.

[11] Chuma M, Nakamoto A, Bando T (2022). Association between statin use and daptomycin-related musculoskeletal adverse events: a mixed approach combining a meta-analysis and a disproportionality analysis. Clin Infect Dis.

[12] Ogura T, Shiraishi C (2024). Analysis of adverse events following phenobarbital administration for pediatric patients categorized by one-year age increments using the U.S. Food and Drug Administration Adverse Event Reporting System. Cureus.

[13] Ogura T, Shiraishi C (2023). Comparison of adverse events occurred during administration of dipeptidyl peptidase-4 inhibitor in patients with diabetes using FDA Adverse Event Reporting System. Clin Drug Investig.

[14] Vickerstaff V, Omar RZ, Ambler G (2019). Methods to adjust for multiple comparisons in the analysis and sample size calculation of randomised controlled trials with multiple primary outcomes. BMC Med Res Methodol.

[15] McHugh ML (2009). The odds ratio: calculation, usage and interpretation. Biochem Med.

[16] Hu D, Wang C, O'Connor AM (2020). A method of back-calculating the log odds ratio and standard error of the log odds ratio from the reported group-level risk of disease. PLoS One.

[17] Deolikar V, Raut SS, Toshniwal S, Kumar S, Acharya S (2024). Navigating the statin landscape: a comprehensive review of stroke prevention strategies. Cureus.

[18] Usman NU, Winson T, Basu Roy P, Tejani VN, Dhillon SS, Damarlapally N, Panjiyar BK (2023). The impact of statin therapy on cardiovascular outcomes in patients with diabetes: a systematic review. Cureus.

[19] Diamond DM, Leaverton PE (2023). Historical review of the use of relative risk statistics in the portrayal of the purported hazards of high LDL cholesterol and the benefits of lipid-lowering therapy. Cureus.

[20] Galicia-Garcia U, Jebari S, Larrea-Sebal A (2020). Statin treatment-induced development of type 2 diabetes: from clinical evidence to mechanistic insights. Int J Mol Sci.

[21] Laakso M, Fernandes Silva L (2023). Statins and risk of type 2 diabetes: mechanism and clinical implications. Front Endocrinol (Lausanne).

[22] Ramkumar S, Raghunath A, Raghunath S (2016). Statin therapy: review of safety and potential side effects. Acta Cardiol Sin.

[23] Rosenberg J, Lampridou S, Moores A, Garfield S, Wingfield D, Judah G (2025). A systematic review uncovering modifiable influences on statin adherence. Patient Prefer Adherence.

[24] Kao DP, Martin JL, Aquilante CL (2024). Sex-differences in reporting of statin-associated diabetes mellitus to the US Food and Drug Administration. BMJ Open Diabetes Res Care.

[25] Faubion SS, Kapoor E, Moyer AM, Hodis HN, Miller VM (2019). Statin therapy: does sex matter?. Menopause.

[26] Zanger UM, Schwab M (2013). Cytochrome P450 enzymes in drug metabolism: regulation of gene expression, enzyme activities, and impact of genetic variation. Pharmacol Ther.

[27] Bakar NS, Neely D, Avery P, Brown C, Daly AK, Kamali F (2018). Genetic and clinical factors are associated with statin-related myotoxicity of moderate severity: a case-control study. Clin Pharmacol Ther.

[28] Marrett E, Zhao C, Zhang NJ (2014). Limitations of real-world treatment with atorvastatin monotherapy for lowering LDL-C in high-risk cardiovascular patients in the US. Vasc Health Risk Manag.

[29] Conforti A, Magro L, Moretti U (2006). Fluvastatin and hepatic reactions: a signal from spontaneous reporting in Italy. Drug Saf.

[30] Adachi K, Ohyama K, Tanaka Y, Saito Y, Shimizu M, Yamazaki H (2024). Modeled hepatic/plasma exposures of fluvastatin prescribed alone in subjects with impaired cytochrome P450 2C9*3 as one of possible determinant factors likely associated with hepatic toxicity reported in a Japanese adverse event database. Biol Pharm Bull.

